# Arbuscular mycorrhizal fungi and rhizobium facilitate nitrogen uptake and transfer in soybean/tobacco intercropping system

**DOI:** 10.3389/fpls.2026.1842437

**Published:** 2026-06-04

**Authors:** Xiaohui Song, Yanlan Xie, Yingang Lu, Xianfeng Hu, Wei Xu, Shouhui Pan, Yuan Xue

**Affiliations:** 1 College of Agriculture, Anshun University, Anshun, Guizhou, China; 2 College of Agriculture, Guizhou University, Guiyang, Guizhou, China; 3 Guizhou Province Tobacco Company Anshun Company, Anshun, Guizhou, China

**Keywords:** arbuscular mycorrhizal fungi, mycorrhizal colonization rate, N transfer, N uptake, rhizobium, soybean/tobacco intercropping

## Abstract

**Introduction:**

The tripartite symbiosis of legume-AMF-rhizobia is widely considered to facilitate nitrogen (N) uptakeby legumes, but its effect on non-legume plants in intercropping systems remains unclear.

**Methods:**

A pot experiment with three root separations (PS, MS, NS) was conducted using ¹⁵N isotope tracing in a soybean/tobacco intercropping system with double inoculation of *Claroideoglomus etunicatum* (CE) and *Bradyrhizobium japonicum* 5016 (BJ).

**Results:**

Double inoculation (CE+BJ) significantly increased bioaccumulation, N uptake, and N transfer from soybean to tobacco compared to single or no inoculation. Mycorrhizal colonization in soybean increased by up to 45.55% in the NS system. All measured parameters in tobacco were significantly higher in NS than in PS or MS systems.

**Discussion:**

The legume-AMF-rhizobium triple interaction positively affects N uptake and translocation in tobacco, showing potential for sustainable tobacco production, though the underlying mechanisms require further study.

## 1 Introduction

Intercropping, a traditional agricultural technique, has garnered significant acclaim for its ability to enhance resource utilization efficiency, augment soil fertility, mitigate adversity caused by heavy metals, escalate crop productivity, and maintain biodiversity and stability of agro-ecosystems (Wang et al., 2024; Chelan et al., 2024; Zhang S. et al., 2024; Massa et al., 2025). Studies confirm intercropping non-N_2_-fixing crops with legumes boosts yields and nutrient uptake. Legumes supply nitrogen via biological N_2_ fixation, creating synergistic advantages in these cropping systems, including wheat-faba bean (Sammama et al., 2022), finger millet-pigeon pea (Singh et al., 2019), potato-climbing bean (Gitari et al., 2020), millet-chickpea (Li et al., 2022), oat−pea (Lee et al., 2023), cassava-legume (Dettweiler et al., 2023), and maize-soybean (Liu et al., 2024). The reported advantages of intercropping with legumes can be explained as follows: (1) The optimal utilization of light, temperature, and space (Dettweiler et al., 2023; Liu et al., 2024); (2) The formation of common mycorrhizal networks (CMNs) among intercropped plant species mediates interplant nutrient complementarity, particularly through enhanced allocation and mycelial bridging mechanisms (Ward et al., 2023; Zhang et al., 2023); (3) Rhizodeposition from leguminous plants enhances nutrient bioavailability and stabilization dynamics within the rhizospheric zone (Zhang S. et al., 2024). However, mechanisms of interspecific N transfer mediated by inter-root interactions between legumes and non-legumes remain inadequately documented.

Arbuscular mycorrhizal fungi (AMF) have the capacity to establish a symbiotic relationship with the majority of vascular plants. The extensive mycelial network formed as a consequence of this symbiosis serves to increase the efficiency of plant nutrient uptake (Zhang et al., 2023; Zhang S. et al., 2024). For example, the powerful underground mycelial network of AMF has been demonstrated to mediate resource partitioning (nutrients and water) among plants, act as a conduit for transmitting disease and aphid-induced signals, and enhance rhizosphere microbial community building through symbiotic interactions (Meyer et al., 2024; Taheri et al., 2025). The aforementioned factors have been demonstrated to reduce nutrient losses, improve disease resistance, enhance resistance to environmental stresses, and increase plant yield and quality (Begum et al., 2021; Du et al., 2023).

Plant growth-promoting rhizobacteria (PGPR) are defined as large numbers of soil bacteria that live freely in the soil or are attached to the rhizosphere of plants. Previous studies have reported that PGPR provides multiple benefits to agroecosystems through symbiotic interactions with host plants. Examples include reducing dependence on chemical fertilizers through biological nitrogen fixation; stimulating root morphogenesis by releasing phytohormones (e.g., auxins, gibberellic acid, and cytokinins), phosphorus (P), and potassium (K); improving water and mineral uptake efficiency (Zhang et al., 2023); producing siderophores and increasing iron and manganese availability (Rezaei‐Chiyaneh et al., 2021); and mitigating abiotic stresses by synthesizing osmolytes (e.g., proline, soluble protein, and soluble sugar), thereby increasing plant tolerance to drought and salinity (Liu et al., 2020; Shang et al., 2023; Zeng et al., 2025).

AMF and PGPR, as typical biofertilisers, not only reduce fertilizer inputs and improve soil fertility, plant growth, yield, and quality (Mathimaran et al., 2020; Yilmaz and Karik, 2022; Slimani et al., 2023; Chen et al., 2023; Agunbiade et al., 2024; Mazumder et al., 2025) but also increase environmental stress (e.g., heavy metal toxicity, drought, salinity, and soil compaction) (Sharma et al., 2021; Karimi and Noori, 2022; Du et al., 2023), decrease the inhibitory effect of plant pathogens (Dere, 2024; Solórzano-Acosta et al., 2024), and promote changes in plant metabolism and modulate nutrient (e.g., nitrogen, phosphorus, and essential minerals) allocation and availability (Tahiri et al., 2022; Najafi Vafa et al., 2022; Zhang et al., 2023; de Azevedo et al., 2024). Studies have shown that N transformation and distribution in soybean/maize intercropping systems are regulated by AMF and PGPR (Qin et al., 2023; Zhang et al., 2023; Parks and Friesen, 2024). This phenomenon can be attributed to the robust underground mycelial network of AMF and the biological nitrogen fixation and additional benefits by PGPR.

Tobacco (*Nicotiana tabacum L.*), a dicotyledonous species within the family Solanaceae, is an economically important crop widely cultivated in China and elsewhere in the world and is readily colonized by AMF (Begum et al., 2021; Cheng et al., 2022; de Leon et al., 2024). Recent studies highlight synergistic AMF-PGPR interactions that optimize plant performance. For example, co-inoculation of AMF and PGPR improved the growth, yield, quality, and stress tolerance of tomato, faba bean, pepper, maize, potato, spinach, basil, satureja, and other plants (Raklami et al., 2019; Khalediyan et al., 2021; Tahiri et al., 2022; Najar et al., 2024; Dere, 2024). In tobacco, some studies have shown that a single inoculation of either AMF or PGPR can, to some extent, promote tobacco seedling growth, increase resistance and improve nutrient uptake (Begum et al., 2020; Liu et al., 2020; Begum et al., 2021; Cheng et al., 2022; Shang et al., 2023; de Leon et al., 2024). Additionally, studies have confirmed that the double inoculation with AMF and PGPR promoted tobacco seedling growth and optimized the structure and function of the rhizosphere microbial community (Begum et al., 2022; Zeng et al., 2025). However, fewer studies have been conducted on the effects of AMF and PGPR co-inoculation on nitrogen uptake and transformation in tobacco/soybean intercropping systems. Therefore, the objective of this study was to ascertain whether the co-inoculation of exogenous AMF and PGPR could enhance nitrogen uptake and transformation when tobacco and soybean (*Glycine max L.cv.*) are intercropped, and to determine how much nitrogen is transformed between the two crops.

## 2 Materials and methods

### 2.1 Experimental materials

In this study, *Claroideoglomus etunicatum* and *Bradyrhizobium japonicum* 5016 were utilized as the AMF and PGPR inoculant, respectively. Among them, CE used the qualified inoculant from 131d propagation of white clover with >50 spores/g, and the BJ strain was cultured in LB medium. The tobacco and soybean varieties selected were Zhongyan 100 and Andou 7, respectively. Soybean seeds come from the Oilseed Institute of Guizhou Academy of Agricultural Sciences and are conventional varieties selected by crossbreeding ZYD05689, a wild bean, with ZDD15633, a local varietal resource. The test soils were then subjected to autoclaving, a process that was intended to eradicate the indigenous AMF and PGPR population. The soil sample selected for the analysis was a yellow soil, which exhibited the following basic physical and chemical properties: water content 27.1%, pH 5.85, organic matter 21.6 g/kg, total nitrogen 1.08 g/kg, total phosphorus 0.75 g/kg, total potassium 11.6 g/kg, alkaline dissolved nitrogen 53.7 mg/kg, and available phosphorus 12.9 mg/kg, available potassium 83.25 mg/kg. The Hoagland nutrient solution comprised the following components: CaNO_3_·4H_2_O 945 mg/L, KNO_3–_506 mg/L, NH_4_NO_3_ 80mg/L, KH_2_PO_4–_136 mg/L, MgSO_4–_493 mg/L, KI 0.83 mg/L, H_3_BO_3_ 6.2 mg/L, MnSO_4_ 22.3 mg/L, ZnSO_4_ 8.6 mg/L, Na_2_MoO_4_ 0.25 mg/L, CuSO_4_ 0.025 mg/L, CoCl_2_ 0.025 mg/L, FeSO_4_·7H_2_O 5.56 g/L、EDTA-Na 7.46 g/L.

### 2.2 Experimental design

A pot experiment was implemented in a greenhouse. The test pots have been fabricated from resin and are of a cylindrical nature (height 23cm, diameter 26cm). Three types of separation were set up in the experiment: Plastic Separate (PS), in which black hard plastic sheets (1mm) were used to separate the potted plants into left and right chambers so that roots, mycelium, water, and nutrients could not pass through; and Mesh separation (MS), in which a 30 μm nylon mesh was used to separate the chambers. Roots could not pass through the chambers, while nutrients, water, and mycelium could pass through the chambers; No separation (NS), no barriers, free passage of roots, mycelium, nutrients, and so forth. Four treatments were used in the experiment: inoculation with *Bradyrhizobium japonicum* 5016 (BJ), inoculation with *Claroideoglomus etunicatum* (CE), double inoculation with *Bradyrhizobium japonicum* 5016+*Claroideoglomus etunicatum* (BJ+CE), and control treatment without inoculum (CT). The 4 × 3 factorial experiments were carried out based on a randomized complete block design with three replications.

The left and right compartments were each filled with 2kg of soil, and the inoculum bacteria/soil was 30 g/kg. The full amount of hoaglands nutrient solution was carefully poured into the compartments, and 40% of the soil water-holding capacity was maintained, while 15 ml of AMF inoculant (>50 spores/g) was thoroughly mixed with the soil. Soybean seeds were soaked in 10% H_2_O_2_ for 5min before sowing and washed with sterilized water, and six soybean seeds were sown into 1/2 compartment, and the number of plants was reduced from 6 to 2 after soybean germination. Twenty days after the soybean seeds were sown, the tobacco seedlings, which had been raised to five leaves and one heart with good growth by the floating seedling method, were transplanted to another compartment. Seven days before the transplanting of the tobacco seedlings, a diluted (800 times) 20% benomyl was poured into the seedling floating solution to eliminate the effect of microorganisms on the root system of the seedlings before transplanting. All pots were marked and randomly placed, and the pots were randomly placed again every fortnight. Sterilized water was added at regular intervals to replenish soil moisture.

The isotope labelling test was carried out using ^15^N-(NH_4_)_2_SO_4_ during soybean pod growth (68 days after transplanting). Before labelling, a PVC plate was inserted between soybean and tobacco to prevent isotopic nitrogen contamination, and two layers of plastic film with filter paper were applied to the soil surface. Ten microliter of ^15^N-(NH_4_)_2_SO_4_ solution at a concentration of 88 mM was injected daily into the lower side of the soybean petiole with a micro syringe (25 μl) for 7 days. Unlabeled plants were used as a control to determine the natural nitrogen abundance. The experiments were carried out for 76 days.

### 2.3 Measurement indices and methods

#### 2.3.1 Determination of mycorrhizal infestation rate

Microscopic observation was used to determine the colonization of AMF (Zhang et al., 2015). Three tobacco plants were randomly selected for each treatment, and the root system was completely extracted. The root system was then rinsed repeatedly with water to obtain the intact root system and then rinsed with deionized water to obtain the young part of the front end of the fibrous root. The root systems were then fixed in 70% FAA standard fixative. The prepared sections were observed, recorded, photographed, and measured using a stereomicroscope (S9 DLeica, Germany) using the square cross-hatch method. Here, mycorrhizal infection rate (%) = (length of infected root segments/length of observed root segments) × 100%.

#### 2.3.2 Determination of soybean and tobacco plant bioaccumulation

After 95d of culture, randomly select the tobacco or soybean plants with uniform growth in each treatment, the whole plants were taken out from the culture pots, and the aboveground and root systems of tobacco or soybean were placed in the oven at 105 C for 30min, dried at 70 C until constant weight, and weighed. Each treatment was repeated 3 times.

#### 2.3.3 Determination of labelled nitrogen ^15^N

Tobacco or soybean Ndff was determined using an elemental analyzer-isotope ratio mass spectrometer (EA-IRMS) (Langelier et al., 2021). Nitrogen transfer was calculated based on Equations 1–4.

(1) N% = N1% − N2%

where N%: atomic percentage of ^15^N in tobacco or soybean, N1%: atomic percentage of ^15^N in labelled plants, N2%: atomic percentage of ^15^N in control plants.

(2) $$\text{Nt}\%\;=\;\left[\left(\text{Nm }\times \text{ Nm}\%\right)/\left(\text{Nm }\times \text{ Nm}\%\;+\text{ Ns }\times \text{ Ns}\%\right)\right]\;\times \;100$$

where Nt% refers to the percentage of nitrogen transferred from the soybean to the relevant tobacco plant, Nm and Ns represent the amount of nitrogen uptake (g/pot) by the tobacco plant and soybean, respectively, and Nm% and Ns%d indicate the percentage of atoms of ^15^N in the tobacco plant and soybean, respectively (based on the control).

(3) Nt = Nt% × Nm

(4) $$\text{No}\%\;=\;\left(\text{Nt}/\text{Nm}\right)\;\times \;100$$

where Nt denotes N transferred from soybean to tobacco plant (mg/pot) and No% expresses N transferred as a percentage of N uptake by the tobacco plant.

### 2.4 Statistical analysis

Data in this study were collated and preliminarily analyzed using Microsoft Excel 2013 and analyzed using two-way ANOVA in SPSS 19.0 SPSS (IBM SPSS Inc., Chicago, IL) software for test analysis (*p*<0.05), and all data measurements are expressed as mean ± standard error. All graphs were done using Origin 8.0 (OriginLab Northampton, MA) software.

## 3 Results and analyses

### 3.1 Effect of inoculation with AMF and rhizobium on colonization rates of tobacco and soybean

**Figure 1:**
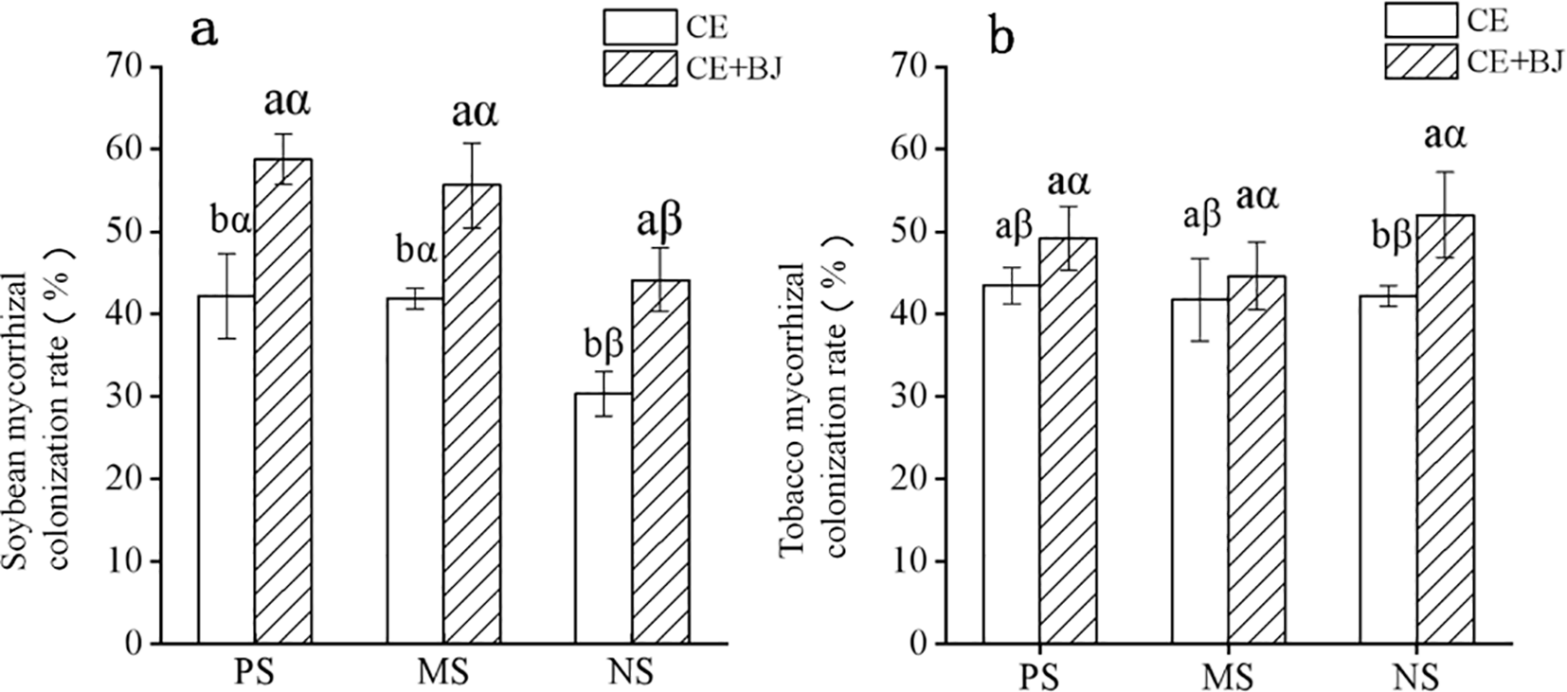
Effect of inoculation with AMF and Rhizobium on infestation rate in soybean **(a)** and tobacco **(b)**. Different inoculation treatments under the same root segregation method are used with lower case letters (a, b, c) to indicate statistical significance (*p*<0.05). Different Greek letters (α, β, γ) indicate statistical significance (*p*<0.05) for different root segregation methods under the same inoculation treatment.

As shown in **Figure 1a**, no AMF colonization was detected in both soybean and tobacco roots not inoculated with CE, and the colonization rates of the unsettled AMF inoculation treatments are not presented for this experiment. The increase in colonization rate was more significant in combined inoculation treatments (CE+BJ) than in single-inoculation treatment (CE). In addition, AMF colonization in PS, MS, and NS systems increased by 39.37%, 32.82%, and 45.55%, respectively, in the soybean colonization rate of CE+BJ double inoculation. In the NS system, the colonization rate of soybean and tobacco was significantly increased in the CE+BJ double inoculation treatment compared to single inoculation with CE. However, there were no statistically significant differences in the PS and MS systems, although tobacco colonization rates were higher in combined CE+BJ inoculation than in CE alone. These results indicated that combined inoculation with AMF and PGPR significantly enhanced mycorrhizal fungal colonization in the root systems of tobacco and soybean plants (*p*<0.05). Furthermore, the AMF colonization rate of tobacco in the NS system was higher than that in the PS mode in both CE and CE+BJ treatments, but not at the level of significant difference (**Figure 1b**).

### 3.2 Effect of inoculation with AMF and rhizobium on bioaccumulation in tobacco and soybean

The total biomass of soybean in PS, MS, and NS systems was increased by 69.98%, 71.09%, and 71.45% in BJ+CE double inoculation treatments compared to CT and 34.20%, 34.18%, and 34.50% in tobacco, respectively (**Table 1**). Additionally, single inoculation of BJ and CE significantly increased aboveground, root, and total bioaccumulation in both soybean and tobacco in three separate systems, respectively, but there were no statistically significant differences between the two. The total bioaccumulation of tobacco in different treatments had the highest value in the NS system, which was significantly greater than the PS and MS systems except for the inoculated BJ treatment, but there was no remarkable impact of different segregation patterns on soybean bioaccumulation, which indicated that intercropping of tobacco with soybean could obviously increase the bioaccumulation of tobacco. Further, no interaction was shown between root isolation and inoculation treatments.

**Table 1:** Above- and below-ground bioaccumulation in tobacco and soybean inoculated with AMF and rhizobacteria(g/pot).

Treatments		Soybean			Tabacco		
		Above-ground	Roots	Total weight	Above-ground	Roots	Total weight
PS	CT	4.32 ± 0.04cα	1.13 ± 0.04cα	5.45 ± 0.07cα	8.49 ± 0.04cγ	5.02 ± 0.06cγ	13.51 ± 0.08cγ
	BJ	5.35 ± 0.02bα	2.19 ± 0.03bα	7.54 ± 0.05bα	9.52 ± 0.02bβ	6.03 ± 0.05bβ	15.55 ± 0.04bβ
	CE	5.39 ± 0.04bα	2.24 ± 0.08bα	7.63 ± 0.11bα	9.51 ± 0.04bβ	6.05 ± 0.03bβ	15.56 ± 0.07bβγ
	BJ+CE	6.68 ± 0.13aα	2.55 ± 0.01aα	9.23 ± 0.05aα	10.82 ± 0.05aβ	7.31 ± 0.05aβ	18.13 ± 0.09aβ
MS	CT	4.27 ± 0.06cα	1.14 ± 0.08cα	5.41 ± 0.13cα	8.61 ± 0.03cβ	5.17 ± 0.03cβ	13.78 ± 0.03cβ
	BJ	5.35 ± 0.02bα	2.25 ± 0.02bα	7.60 ± 0.04bα	9.62 ± 0.02bβ	6.25 ± 0.03bα	15.87 ± 0.06bα
	CE	5.45 ± 0.10bα	2.27 ± 0.04bα	7.72 ± 0.07bα	9.67 ± 0.02bβ	6.22 ± 0.02bα	15.89 ± 0.05bβ
	BJ+CE	6.74 ± 0.07aα	2.55 ± 0.12aα	9.29 ± 0.08aα	10.96 ± 0.15aα	7.53 ± 0.03aα	18.49 ± 0.06aαβ
NS	CT	4.20 ± 0.08cα	1.23 ± 0.05cα	5.43 ± 0.06cα	8.70 ± 0.05cα	5.27 ± 0.02cα	13.97 ± 0.09cα
	BJ	5.37 ± 0.03bα	2.24 ± 0.04bα	7.61 ± 0.06bα	9.81 ± 0.05bα	6.31 ± 0.03bα	16.12 ± 0.06bα
	CE	5.34 ± 0.06bα	2.17 ± 0.06bα	7.51 ± 0.14bα	9.89 ± 0.06bα	6.34 ± 0.04bα	16.23 ± 0.07bα
	BJ+CE	6.77 ± 0.10aα	2.54 ± 0.04aα	9.31 ± 0.08aα	11.13 ± 0.16aα	7.66 ± 0.10aα	18.79 ± 0.22aα
Inoculation		*	*	*	**	**	**
Separation		ns	ns	ns	**	**	**
Inoculation × Separation		ns	ns	ns	Ns	ns	ns

The above data are expressed as mean ± standard deviation (*n*=3). Different inoculation treatments under the same root segregation method using lower case letters (a, b, c) indicate statistical significance (p<0.05). Different Greek letters (α, β, γ) indicate statistical significance (*p*<0.05) for different root segregation methods under the same inoculation treatment. ns indicates no significant difference; * and ** are statistically significant at 0.05 and 0.01 levels, respectively.

### 3.3 Effect of inoculation with AMF and rhizobium on nitrogen concentration and nitrogen uptake in tobacco and soybean

**Figure 2:**
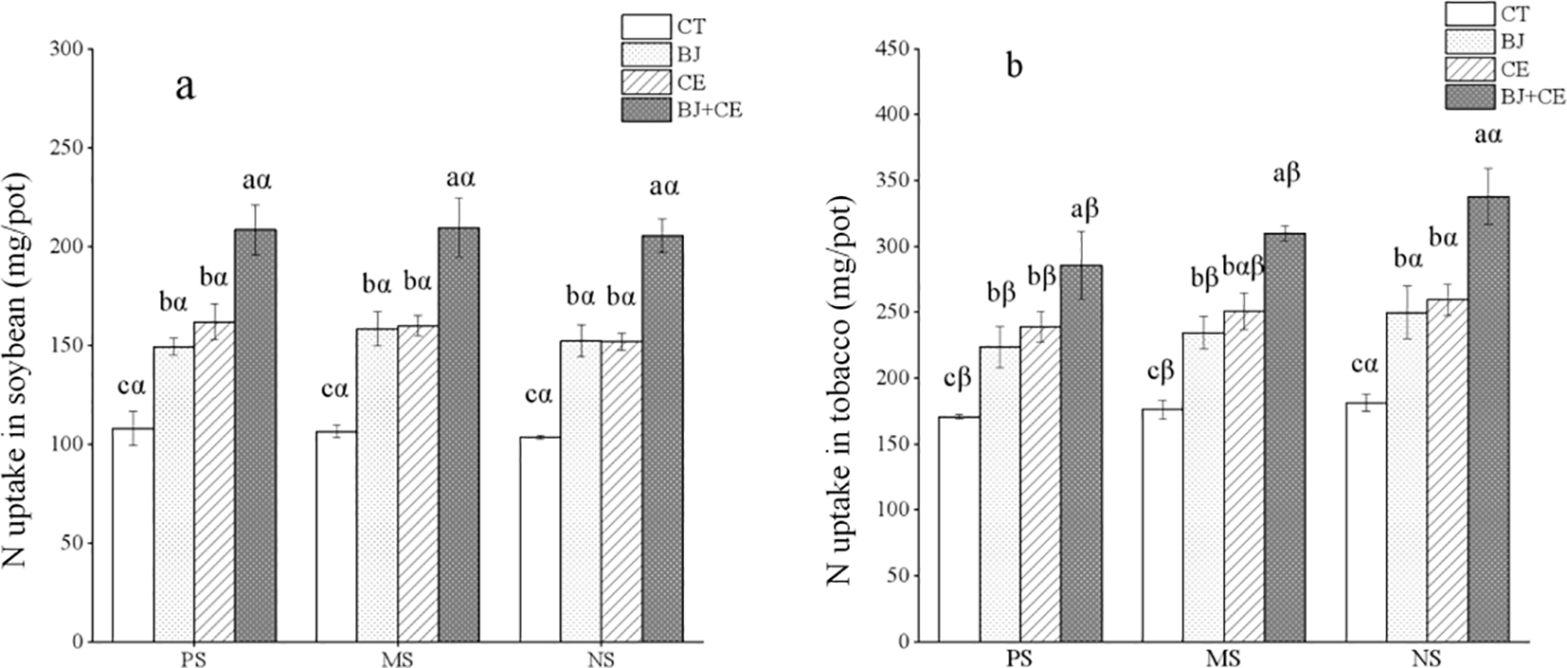
Effect of inoculation with AMF and Rhizobium on nitrogen uptake in tobacco **(a)** and soybean **(b)**. Different inoculation treatments under the same root segregation method are used with lower case letters (a, b, c) to indicate statistical significance (*p*<0.05). Different Greek letters (α, β, γ) indicate statistical significance (*p*<0.05) for different root segregation methods under the same inoculation treatment.

The aboveground and root N concentrations and N absorption in tobacco (13.56%, 73.62%, 20.72%, 173.46%) and soybean (25.04%, 59.10%, 23.19%, 78.66%) were substantially higher in the double-inoculated CE+BJ treatments than in CT, as illustrated in **Table 2**, in all three segregation patterns. Subsequently, N absorption in the aboveground, roots of tobacco and soybean was enhanced in double-inoculated CE+BJ treatments in PS, MS, and NS systems compared to single inoculation with CE or BJ. N concentration and N absorption in the aboveground and roots of soybean were not significantly affected by the different separation methods. However, this was not observed for tobacco, whose N absorption was significantly more than that in both MS and PS systems for different inoculation treatments in the NS system, and there was not a statistically significant difference between the MS and PS systems. Total nitrogen absorbed by tobacco and soybean had the same pattern as aboveground and root (**Figure 2**). In addition, total N acquisition in soybean and tobacco was considerably higher than CT in all inoculation treatments, with the double inoculation CE+BJ treatment having the greatest N acquisition, which was noticeably greater than single inoculation CE or BJ, and there was no significant difference between single inoculation CE and BJ. These results revealed that either single or double inoculation with CE and BJ promoted N uptake in both soybean and tobacco, and secondly, intercropping was more favorable to enhance N acquisition in tobacco.

**Table 2:** Effect of AMF and rhizobium inoculation on nitrogen concentration (mg/kg) and nitrogen uptake (mg/pot) of tobacco and soybean.

Treatments		Soybean				Tabacco			
		Above-ground		Roots		Above-ground		Roots	
		Nitrogen concentrations	Nitrogen absorption	Nitrogen concentrations	Nitrogen absorption	Nitrogen concentrations	Nitrogen absorption	Nitrogen concentrations	Nitrogen absorption
PS	CT	19.91 ± 0.50bα	86.86 ± 1.46cα	18.87 ± 0.80cα	21.06 ± 1.36cα	11.78 ± 0.50cβ	99.87 ± 4.20cβ	14.1 ± 0.20cα	71.01 ± 1.44cβ
	BJ	19.94 ± 0.20bα	106.30 ± 1.15bcα	19.74 ± 0.60bcα	42.96 ± 1.15bα	13.77 ± 0.30bβ	130.88 ± 2.69cβ	15.39 ± 0.40bα	92.72 ± 1.92bβ
	CE	21.18 ± 0.40abα	113.56 ± 1.84bα	21.71 ± 0.80abα	48.33 ± 2.91bα	15.27 ± 0.10aβ	145.21 ± 1.76bβ	15.46 ± 0.20bα	93.52 ± 1.93bβ
	BJ+CE	22.61 ± 0.80aα	150.81 ± 5.37aα	22.78 ± 0.90aα	57.59 ± 2.43aα	14.73 ± 0.50abβ	158.89 ± 5.62aβ	17.37 ± 0.10aβ	126.87 ± 1.79aβ
MS	CT	19.69 ± 0.40bα	84.76 ± 1.76cα	18.90 ± 0.20cα	21.49 ± 5.30cα	11.99 ± 0.40bβ	103.02 ± 2.99cβ	14.22 ± 0.10cα	73.23 ± 0.91cβ
	BJ	20.76 ± 1.00abα	111.52 ± 5.35bα	20.92 ± 0.40bα	46.79 ± 1.15bα	14.27 ± 0.50aβ	137.54 ± 5.41bβ	15.56 ± 0.20bα	96.98 ± 0.95bβ
	CE	20.26 ± 1.20abα	110.33 ± 6.48bα	21.57 ± 0.50bα	49.48 ± 1.41bα	15.89 ± 0.50aαβ	153.04 ± 5.68aα	15.71 ± 0.20bα	97.68 ± 1.34bβ
	BJ+CE	22.50 ± 0.30aα	151.28 ± 2.84aα	22.80 ± 0.20aα	58.21 ± 1.15aα	15.34 ± 1.00aαβ	167.89 ± 10.70aαβ	18.85 ± 0.30aαβ	141.93 ± 2.54aα
NS	CT	19.59 ± 0.30bα	82.46 ± 1.72cα	18.79 ± 0.30cα	21.10 ± 0.85cα	13.23 ± 0.20cα	115.08 ± 1.65dα	14.49 ± 0.50bα	76.37 ± 2.26cα
	BJ	20.20 ± 0.50bα	108.25 ± 2.89bα	19.88 ± 0.10bcα	43.85 ± 0.63bα	15.24 ± 0.10bα	149.67 ± 0.68cα	15.89 ± 0.80bα	100.06 ± 4.91bα
	CE	19.92 ± 0.70bα	106.52 ± 4.08bα	20.71 ± 0.70bcα	45.25 ± 0.53bα	16.08 ± 0.10bα	159.14 ± 1.87bα	15.84 ± 0.60bα	100.18 ± 3.79bα
	BJ+CE	22.34 ± 0.50aα	149.48 ± 5.23aα	22.27 ± 0.30aα	55.98 ± 1.59aα	16.98 ± 0.20aα	188.31 ± 2.26aα	19.54 ± 0.80aα	149.61 ± 6.11aα
Inoculation		*	*	*	**	*	*	*	**
Separation		Ns	ns	ns	ns	ns	ns	ns	**
Inoculation × Separation		Ns	ns	ns	ns	ns	ns	ns	Ns

The above data are expressed as mean ± standard deviation (n=3). Different inoculation treatments under the same root segregation method using lower case letters (a, b, c) indicate statistical significance (*p*<0.05). Different Greek letters (α, β, γ) indicate statistical significance (*p*<0.05) for different root segregation methods under the same inoculation treatment. ns indicates no significant difference; * and ** are statistically significant at 0.05 and 0.01 levels, respectively.

### 3.4 Effect of inoculation with AMF and rhizobium on nitrogen transfer in a soybean/tobacco intercropping system

All inoculation treatments in the soybean/tobacco intercropping system promoted N transfer from soybean to tobacco in both MS and NS systems compared to CT (**Table 3**). Nt increased by 207.43% and 158.32% over CT in double-inoculated CE+BJ treatments in MS and NS systems, respectively. Nt was also substantially higher in the double-inoculated CE+BJ treatment than in single-inoculated BJ or CE in both MS and NS modes and remained non-significantly different between single-inoculated BJ and CE treatments. This study also identified that N transfer from soybean to tobacco accounted for 3.13% to 6.01% of the total N uptake by tobacco in both MS and NS systems and was markedly larger in combined inoculated treatments than in non-inoculated treatments. What was more interesting was that Nt%, Nt, and No were significantly greater in NS systems than in MS systems. This implied that intercropping with soybean coupled with double inoculation with AMF and PGPR significantly increased N uptake in tobacco.

**Table 3:** ^15^N content of AMF and Rhizobium inoculated soybean transferred to tobacco based on reticulated segregation and no segregation patterns.

Treatments	Nt %		Nt (mg/pot)		No %	
	MS	NS	MS	NS	MS	NS
CT	5.18cβ	7.57cα	5.52cβ	7.84cα	3.13cβ	4.10bα
BJ	6.29bβ	8.36bα	9.97bβ	12.72bα	4.25bβ	5.09aα
CE	6.08bβ	9.16bα	9.72bβ	13.91bα	3.89bβ	5.36aα
BJ+CE	8.10aβ	9.88aα	16.97aβ	20.30aα	5.48aβ	6.01aα

The above data are expressed as mean ± standard deviation (*n*=3). Different lowercase letters (a, b, c) were used to indicate statistical significance (*p*<0.05) for different inoculation treatments under the same root segregation method. Different Greek letters (α, β, γ) indicate statistical significance (*p*<0.05) for different root segregation methods under the same inoculation treatment.

## 4 Discussion

The level of mycorrhizal colonization rate partly reflected the improvement of host plant growth and biotic or abiotic stress by AMF (Wu et al., 2024; Yang et al., 2025; Zeng et al., 2025). Rising evidence showed that combined AMF and PGPR inoculation significantly increased the colonization rate of AMF in host plant roots, resulting in improved soil fertility, plant growth, and enhanced plant biomass accumulation (Barzegari Barogh et al., 2023; Chen et al., 2023; Chatzistathis et al., 2024; Yilmaz, 2025). It was observed, for instance, that co-inoculation with *Pseudomonas* sp.SG29 and *Pseudomonas* sp.SG42 boosted the colonization of *F. mosseae* in tobacco roots with significantly higher colonization rates of 53.15% and 45.11%, respectively, compared to inoculation of *F. mosseae* alone (Zeng et al., 2025). This outcome is consistent with the results of a related study where combined AMF and PGPR inoculation significantly increased the mycorrhizal colonization rate of AMF in barley and alfalfa. Furthermore, this study also identified that AMF and PGPR co-inoculation resulted in the highest mycorrhizal colonization rate in a barley and alfalfa intercropping system compared to monoculture (Slimani et al., 2023). It indicated that intercropping with AMF+PGPR co-inoculation is more conducive to promoting plant growth. However, conflicting evidence has been reported that double inoculation with *F. mosseae* and *Bacillus* did not significantly increase the AMF colonization rate of *Elymus nutans* Griseb roots compared with single *F. mosseae* inoculation (Yu et al., 2022). A comprehensive review of available literature revealed that AMF and rhizobacterial co-inoculation had various effects, including positive (Liu et al., 2023; Yu et al., 2024), negative (Tsikou et al., 2022), and no effect on plant root colonization (Zhou et al., 2022). This can be explained by the fact that the mechanism of interaction between AMF and PGPR varied for different microorganisms, host plants, cultivation methods, and growth environments (Gorgia and Tsikou, 2025). In the present study, the mycorrhizal colonization rate of both tobacco and soybean roots co-inoculated with CE+BJ in the NS system was significantly higher than the CE single inoculation treatment. More interestingly, the mycorrhizal colonization rate of tobacco was significantly greater than soybean in the NS system (**Figure 1**). Further, mycorrhizal colonization was significantly lower (*p*<0.05) in double-inoculated AMF and Rhizobium soybeans in the NS system than in the PS and MS systems. This indicated that intercropping coupled with CE+BJ combined inoculation was more favorable to promote AMF colonization in tobacco roots. This result was also confirmed by another study (Massa et al., 2025). Thus, the synergistic effect of CE and BJ had a positive impact on the host plant. However, the underlying mechanisms of rhizobium-AMF interactions and effects needed to be further explored.

AMF and PGPR, as plant growth-promoting microorganisms, have been proved to facilitate plant nutrient availability, improve soil structure, and alleviate environmental stresses, thereby supporting plant growth and health, which makes them valuable in sustainable agricultural practices (Taheri et al., 2025). As our results illustrated, aboveground, root, and total bioaccumulation for tobacco and soybean were significantly higher in mono- or dual-inoculated CE and BJ treatments than in uninoculated treatments (CT) in three different separation systems. Additionally, aboveground, root, and total bioaccumulation of tobacco were significantly higher in the NS system than in the PS system, but there was no significant effect of different segregation on soybean aboveground, root, and total bioaccumulation, which confirmed that intercropping with soybeans significantly enhanced tobacco’s bioaccumulation. Combining intercropping and AMF+PGPR co-inoculation improved total bioaccumulation by 70.83% in soybean and by 39.08% in tobacco, respectively, compared to the controls. This might be attributed to intercropping’s increased root traits space, which in turn enhanced nutrient use efficiency and improved growth performance of intercropped plants (Slimani et al., 2023; Zhang S. et al., 2024; Mechri et al., 2024). In our study, the most pronounced increase in tobacco root N uptake was found in NS. It was further evidenced that intercropping with legumes significantly increased tobacco nutrient uptake efficiency and bioaccumulation.

Legumes-nitrogen-fixing rhizobacteria-AMF have the ability to establish a tripartite symbiotic relationship, which was found to enhance plant biomass, root growth, and root nitrogen content (Yu et al., 2024; Mugabo et al., 2024; Gorgia and Tsikou, 2025). Our results revealed that the mycorrhizal colonization rate of soybean in the CE+BJ co-inoculation treatment was significantly higher than in the CE single inoculation, which confirmed that BJ had a facilitative effect on mycorrhizal colonization of soybean. Further, nitrogen uptake by both roots and shoots was significantly increased in CE+BJ double inoculation compared to BJ sole inoculation, which was hypothesized to be possibly attributed to AMF colonization of soybean roots that had a positive effect on root nodule symbiosis and hence promoted the air or soil N fixation by rhizobial symbiosis. It has been documented that AMF hyphae facilitated rhizobial dispersal and nodulation in legumes (Liu et al., 2023; He et al., 2024) and has a positive effect on rhizobial nitrogenase activity (Zhou et al., 2022; Tsikou et al., 2022; Zhang F. et al., 2024; Yu et al., 2024). However, there were some conflicting reports; for example, in one study, it was documented that dual inoculation with AMF and rhizobia had no effect on nitrogen fixation and nodule dry weight of *Vicia faba* in a field study (Sánchez-Navarro et al., 2020), while the results of another study showed that both AMF and rhizobacteria double inoculation had a negative effect on *Vicia faba* nodule number in a greenhouse pot experiment (Pereira et al., 2019). This may be associated with the identity of the partner and its compatibility environmental conditions such as light limitation and nutrient availability (Gorgia and Tsikou, 2025). Nevertheless, current studies have not succeeded in deciphering the mechanisms of interactions between legume-AMF-rhizobia, especially what mechanisms are involved in the antagonism between rhizobia and AMF.

In this experiment, we found that a facilitated process occurs between soybean and tobacco, as evidenced by tobacco biomass and N uptake being significantly greater than that of soybean in all root separation patterns, regardless of the status of inoculation. In addition, tobacco biomass and N uptake from soybean were significantly greater in NS systems than in MS systems (**Table 3**), which indicated that intercropped treatments resulted in increased tobacco growth and N uptake, as well as mycorrhizal colonization rate, which was most pronounced in the no-separation system. This could be ascribed to symbiotic N fixation from legumes that may be transported to tobacco in intercropping systems either directly through the mycorrhizal network or indirectly through root exudates or rhizosphere deposition (Massa et al., 2025; Gorgia and Tsikou, 2025). More interestingly, we also observed that Nt%, Nt, and No% in AMF and Rhizobium co-inoculation treatments were significantly enhanced in the NS system as compared to the MS system (**Table 3**). This may be interpreted as soybean-tobacco root-to-root interactions in intercropping systems driving N transfer from soybean to tobacco. Particularly, the tripartite legume-AMF-rhizobium interaction contributed greatly to facilitating nitrogen uptake in tobacco (Gorgia and Tsikou, 2025). For instance, the extraradical mycelium formed by AMF with legumes promoted rhizobia to migrate long distances to the roots to form rhizomes, which resulted in increased N fixation in soil or air by legumes. Simultaneously, the extraradical mycelium attached rhizobia, legumes, and non-legumes together, and this attachment greatly fostered N uptake by non-legumes (He et al., 2024). One study revealed that AMF symbiosis could mediate the accumulation of rhizobia in the rhizosphere of mycorrhizal plants and hence stimulate the rhizobium-legume symbiosis (Wang et al., 2021). Another study demonstrated the presence of legume-AMF-rhizobium interactions in intraradical roots (Tsikou et al., 2022). Integrated studies suggest that rhizobacterial and AMF interactions involve both intraradical and extraradical roots (Gorgia and Tsikou, 2025). Unfortunately, key information on the regulation of N uptake and translocation mechanisms by the legume-AMF-rhizobium symbiosis has remained lacking, and more basic research will be needed to unravel the tripartite interactions and investigate this symbiotic relationship’s potential for application in sustainable agricultural practices before we can utilize this tripartite system to ameliorate environmental stresses and solve agricultural problems.

## 5 Conclusion

In this study, we observed that all inoculation treatments in three separation systems significantly increased aboveground and root bioaccumulation and N uptake in tobacco and soybean. Among them, co-inoculation with AMF and rhizobium treatments yielded the best results in terms of some indicators (mycorrhizal colonization rate, bioaccumulation, N absorption, Nt%, Nt, and No). Among three separation systems, mycorrhizal colonization of soybean in CE+BJ double inoculation treatment was significantly higher than CE single inoculation, and we speculated that there was a positive effect of rhizobium on mycorrhizal colonization of soybean. However, the aboveground and root bioaccumulation and N uptake of soybean were not affected by various root segregation methods. Conversely, tobacco bioaccumulation, N uptake, and transport were significantly greater in the NS system than in the MS and PS systems. This evidenced that intercropping with soybean coupled with co-inoculation with AMF and Rhizobium was preferred to boost N use efficiency in tobacco. These results illustrated dual AMF-Rhizobium inoculation and intercropping synergies have certain potentials in alleviating environmental stresses (e.g., N deficiency) and enhancing agricultural sustainability. Nevertheless, the underlying mechanisms of the legume-AMF-rhizobium triple interaction effects on host plants need to be further investigated.

## Data Availability

The original contributions presented in the study are included in the article/supplementary material. Further inquiries can be directed to the corresponding author.
